# Supplementary material for: Brain tissue oxygen pressure combined with intracranial pressure monitoring may improve clinical outcomes for patients with severe traumatic brain injury: a systemic review and meta-analysis
Source: PeerJ. 2024 Oct 8;12:e18086. doi: 10.7717/peerj.18086 (PMC11468803; doi:10.7717/peerj.18086)
Supplement: Supplemental Information 2 [file peerj-12-18086-s002.docx]

**Supplementary Material 2: Searching strategies and parameters for trial sequential analysis**

**Searching strategies**

#1 ((((((((((((((((((((head trauma[Title/Abstract]) OR head injury[Title/Abstract]) OR traumatic cerebral injury[Title/Abstract]) OR cerebral injury[Title/Abstract]) OR traumatic brain insult[Title/Abstract]) OR brain lesion, traumatic[Title/Abstract]) OR brain system trauma[Title/Abstract]) OR brain trauma[Title/Abstract]) OR cerebral trauma[Title/Abstract]) OR encephalopathy, traumatic[Title/Abstract]) OR mild traumatic brain injury[Title/Abstract]) OR organic cerebral trauma[Title/Abstract]) OR posttraumatic encephalopathy[Title/Abstract]) OR traumatic brain injuries[Title/Abstract]) OR traumatic brain injury[Title/Abstract]) OR traumatic brain lesion[Title/Abstract]) OR traumatic encephalopathy[Title/Abstract]))

AND

#2 (((((((((brain oxygen[Title/Abstract]) OR Brain Tissue Oxygen Monitoring[Title/Abstract]) OR Brain Oxygen Monitoring[Title/Abstract]) OR cerebral Oxygen Monitoring[Title/Abstract]) OR brain tissue oxygen tension monitoring[Title/Abstract]) OR pBtO2 monitor*[Title/Abstract]) OR pBtO2[Title/Abstract]) OR Licox[Title/Abstract]) OR Brain Tissue Oxygen[Title/Abstract])))

**Trial Sequential Analysis**

The following parameters for trial sequential analysis (TSA) were pre-specified: alpha 5%, beta 10% (power 90%), and the DerSimonian–Laird random effect model. Between-trial heterogeneity was adjusted by the diversity-estimate. A continuity correction factor of 0.5 was added in case of zero events. The effect sizes for categorical outcomes (favourable neurological outcome at 6 months, in-hospital mortality, long-term mortality) were 10%, and this was based on the clinically meaningful and realistic magnitude. For continuous outcomes (length of stay in intensive care unit, length of stay in hospital), the minimal important difference was based on the clinically meaningful and realistic magnitude. For categorical outcomes, the proportion of events in the control population was based on the pooled observed event rate of the updated meta-analysis. For continuous outcomes, the variance was based on the pooled observed standard deviation of the current updated meta-analysis^1^. This approach will maximize the generalizability of the results.

1. Santana LS, Diniz JBC, Solla DJF, et al. Brain tissue oxygen combined with intracranial pressure monitoring versus isolated intracranial pressure monitoring in patients with traumatic brain injury: an updated systematic review and meta-analysis. *Neurol Sci* 2024 doi: 10.1007/s10072-024-07392-0 [published Online First: 2024/02/14]
