# Supplementary material for: Brain tissue oxygen pressure combined with intracranial pressure monitoring may improve clinical outcomes for patients with severe traumatic brain injury: a systemic review and meta-analysis
Source: PeerJ. 2024 Oct 8;12:e18086. doi: 10.7717/peerj.18086 (PMC11468803; doi:10.7717/peerj.18086)
Supplement: Supplemental Information 3 [file peerj-12-18086-s003.docx]

**Supplementary material 3**

**Table 1 The quality assessment of included studies by the Cochrane risk of bias tool**

| Study | Cochrane risk of bias tool components | | | | | | |
| --- | --- | --- | --- | --- | --- | --- | --- |
|  | 1 | 2 | 3 | 4 | 5 | 6 | 7 |
| Payen 2023 (OXY-TC trial) | - | - | + | - | - | - | - |
| Okonkwo 2017 (BOOST-II trial) | - | - | + | - | - | - | - |
| Lin 2015 | ? | ? | + | ? | - | - | - |
| Lee 2010 | - | - | - | - | - | - | - |

1: Random sequence generation; 2: Allocation concealment; 3: Blinding of participants and personnel; 4: Blinding of outcome assessment; 5: Incomplete outcome data; 6: Selective reporting; 7: Other bias.

“-” indicates low risk of bias, “+” indicates high risk of bias, “?” indicates unclear risk of bias.

**Table 2 The quality assessment of included studies by the Newcastle-Ottawa Scale for cohort studies**

| Study | Newcastle-Ottawa Scale components | | | | | | | | Quality score |
| --- | --- | --- | --- | --- | --- | --- | --- | --- | --- |
|  | 1 | 2 | 3 | 4 | 5 | 6 | 7 | 8 |  |
| Roman 2023 | * | * | * | * |  | * | * | * | 7 |
| Barrit 2022 | * | * | * | * | ** | * | * |  | 8 |
| Hoffman 2021 | * | * | * | * | ** | * | * | * | 9 |
| Sekhon 2017 | * | * | * | * |  | * | * | * | 7 |
| Green 2013 | * | * | * | * |  | * | * | * | 7 |
| Spiotta 2010 | * | * | * | * |  | * | * | * | 7 |
| Mccarthy 2009 | * | * | * | * |  | * | * | * | 7 |
| Narotam 2009 | * | * | * | * |  | * | * | * | 7 |
| Martini 2009 | * | * | * | * |  | * | * | * | 7 |
| Adamides 2009 | * | * | * | * | ** | * | * | * | 9 |
| Stiefel 2005 | * | * | * | * |  | * | * | * | 7 |
| Meixensberger 2003 | * | * | * | * |  | * | * | * | 7 |

1: Representativeness of the exposed cohort; 2: Selection of the non-exposed cohort; 3: Ascertainment of exposure; 4: Demonstration that outcome of interest was not present at start of study; 5: Comparability of cohorts on the basis of the design or analysis; 6: Assessment of outcome; 7: Was follow-up long enough for outcomes to occur; 8: Adequacy of follow up of cohorts.
