# Supplementary material for: Brain tissue oxygen pressure combined with intracranial pressure monitoring may improve clinical outcomes for patients with severe traumatic brain injury: a systemic review and meta-analysis
Source: PeerJ. 2024 Oct 8;12:e18086. doi: 10.7717/peerj.18086 (PMC11468803; doi:10.7717/peerj.18086)
Supplement: Supplemental Information 4 [file peerj-12-18086-s004.docx]

**Supplementary Material 4:** Publication bias assessment by funnel plot and Egger’s test, sensitivity analyses.


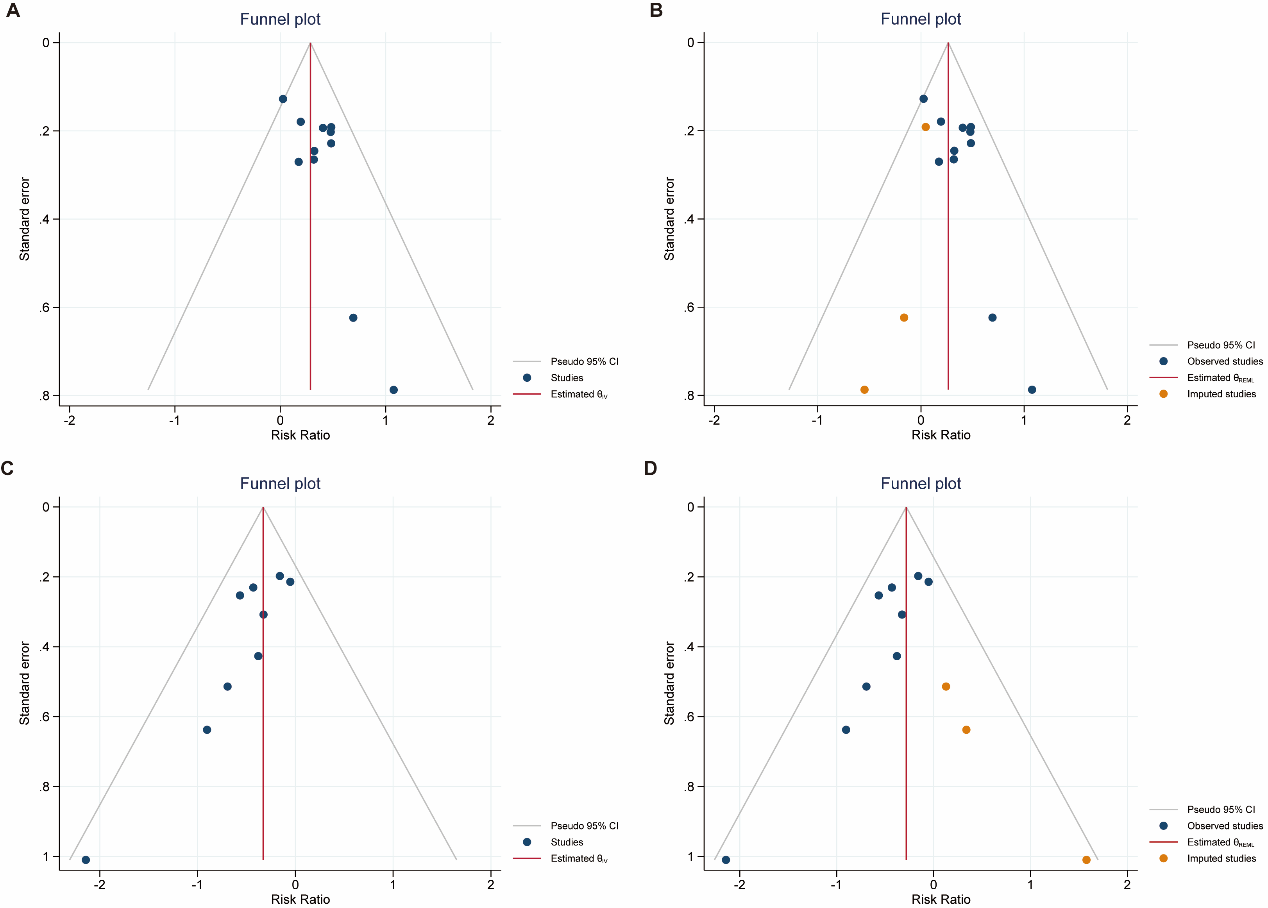


Figure 1: Funnel plot for (A) favourable neurological outcome, Egger’s test P=0.033; (B) favourable neurological outcome after trim-and-fill method (RR: 1.30, 95% CI 1.14 to 1.49); (C) long-term mortality, Egger’s test P=0.011; (D) long-term mortality after trim-and-fill method (RR: 0.75, 95% CI 0.63 to 0.91);


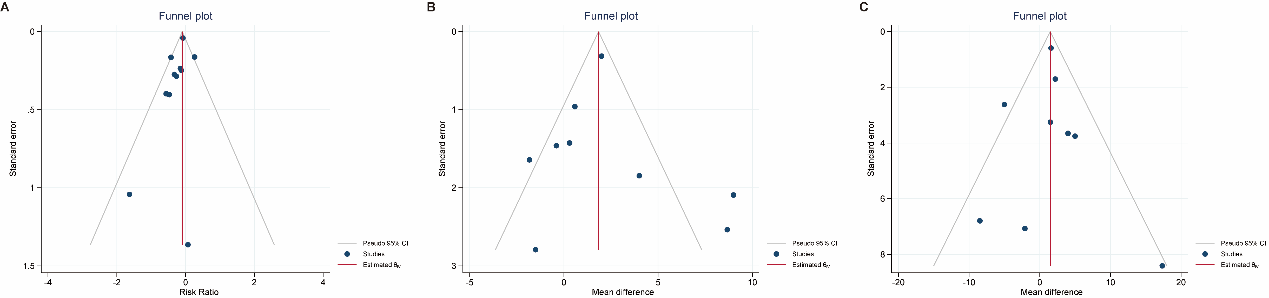


Figure 2: (A) short-term mortality, Egger’s test P=0.144; (B) length of stay in ICU, Egger’s test P=0.919; (C) length of stay in hospital, Egger’s test P=0.896


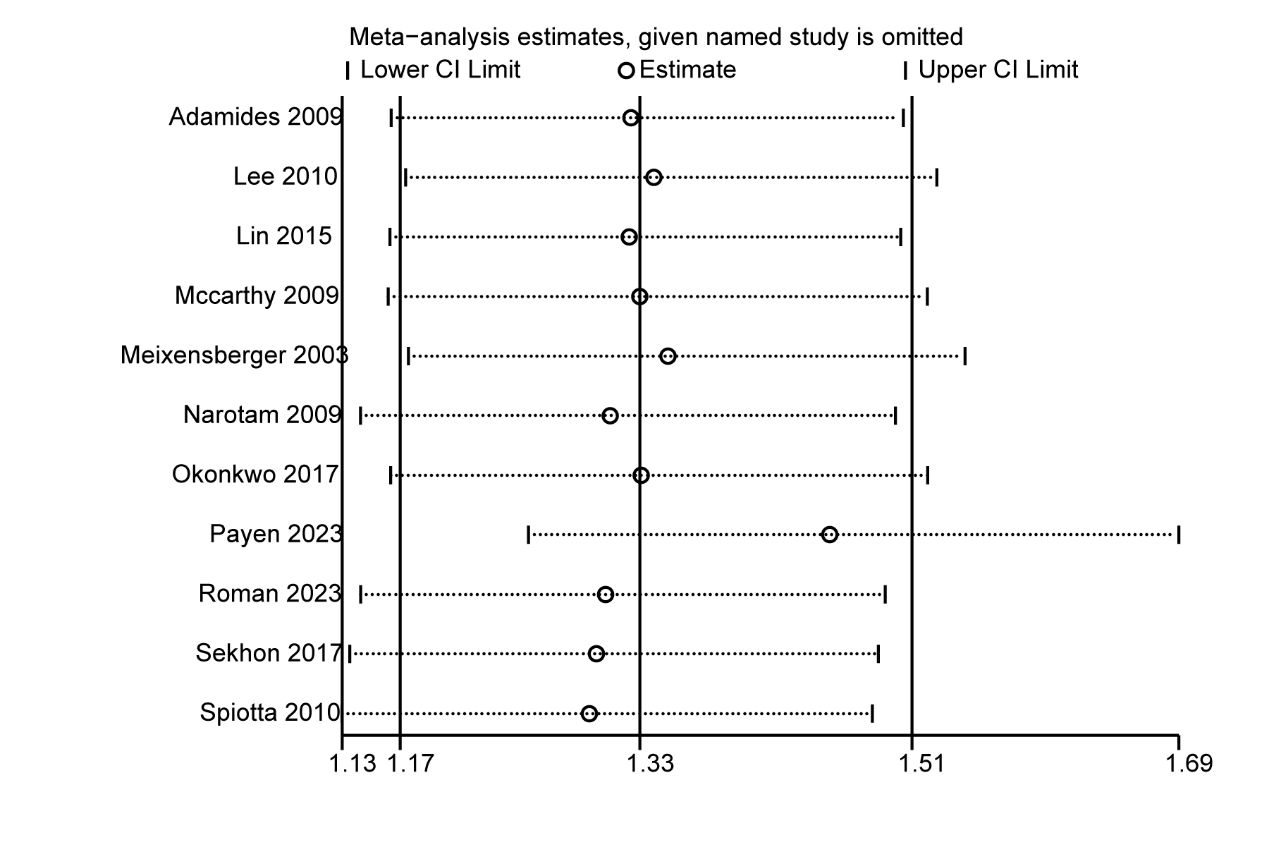
Figure 3: Sensitivity analysis for favourable neurological outcome


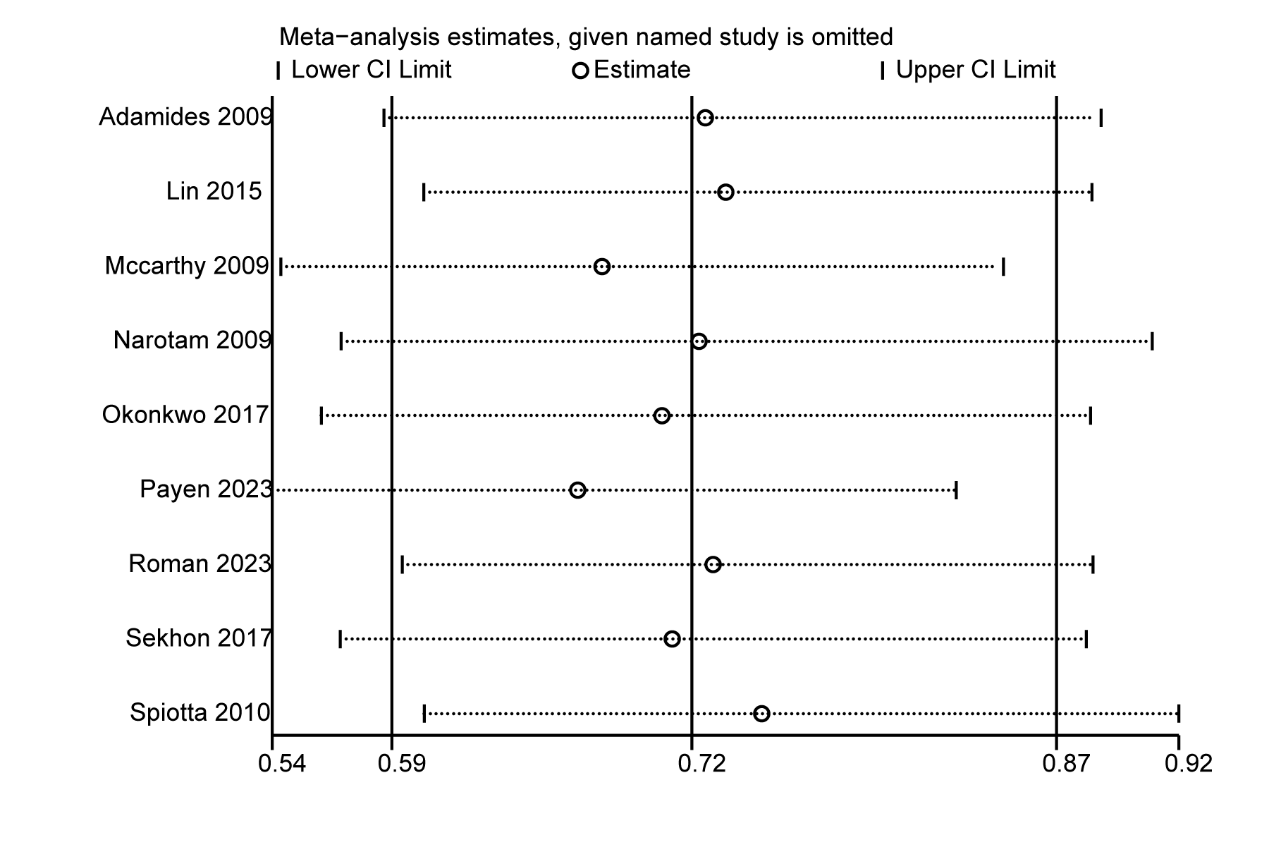


Figure 4: Sensitivity analysis for long-term mortality


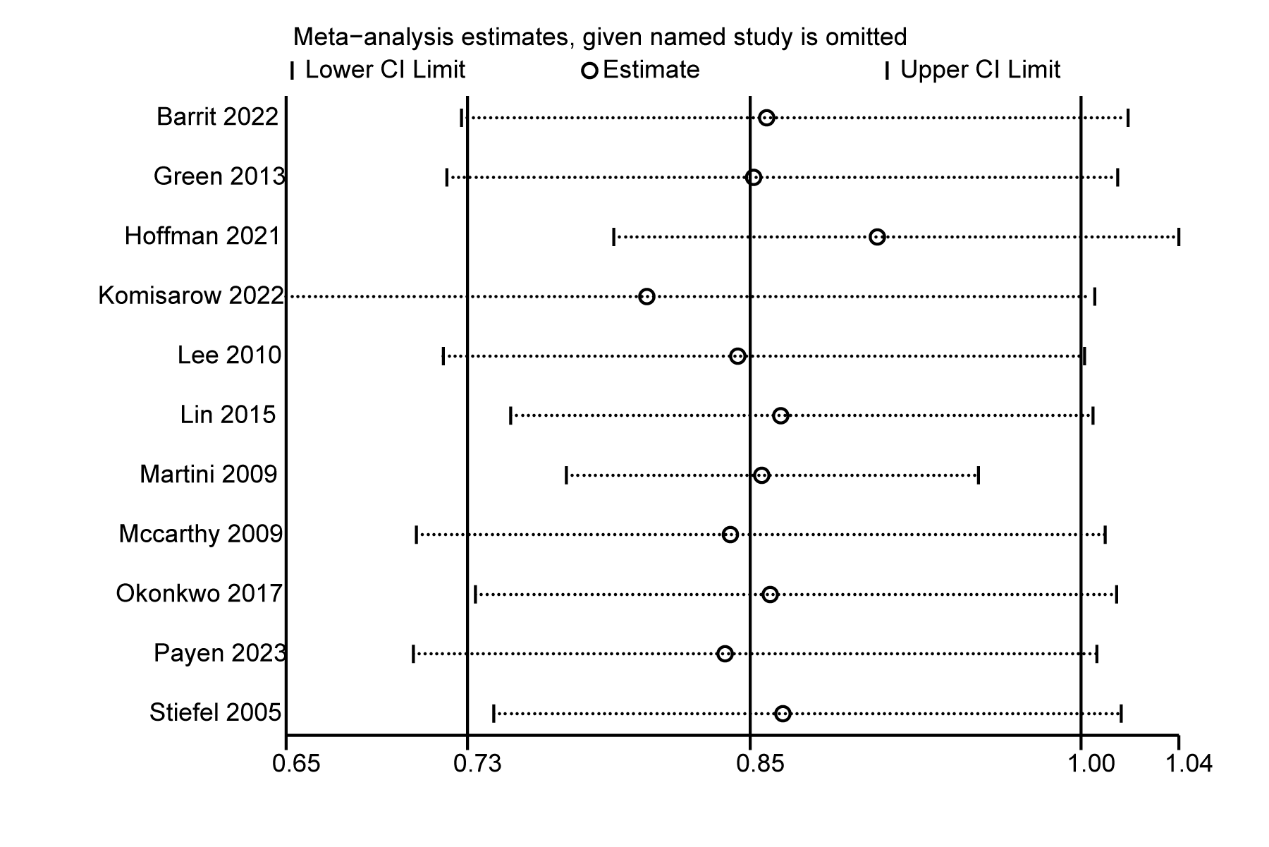


Figure 5: Sensitivity analysis for short-term mortality


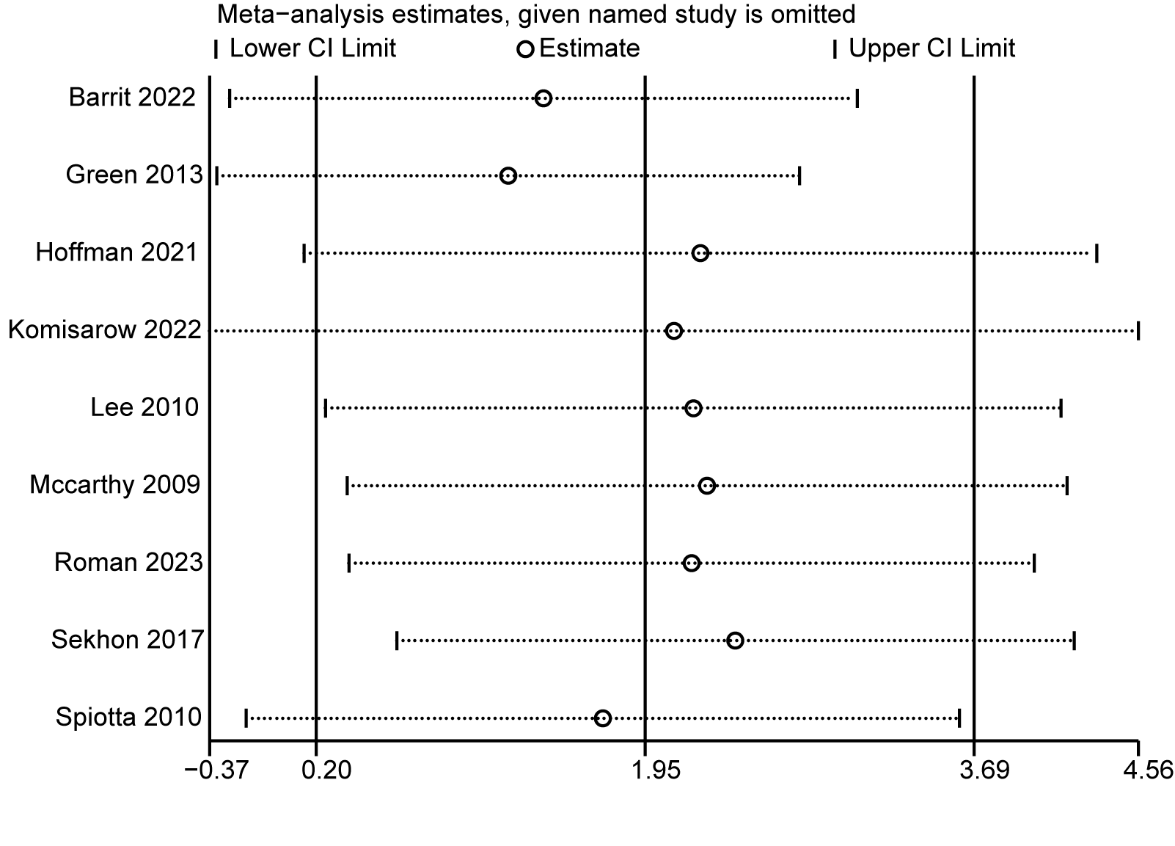


Figure 6: Sensitivity analysis for length of ICU stay


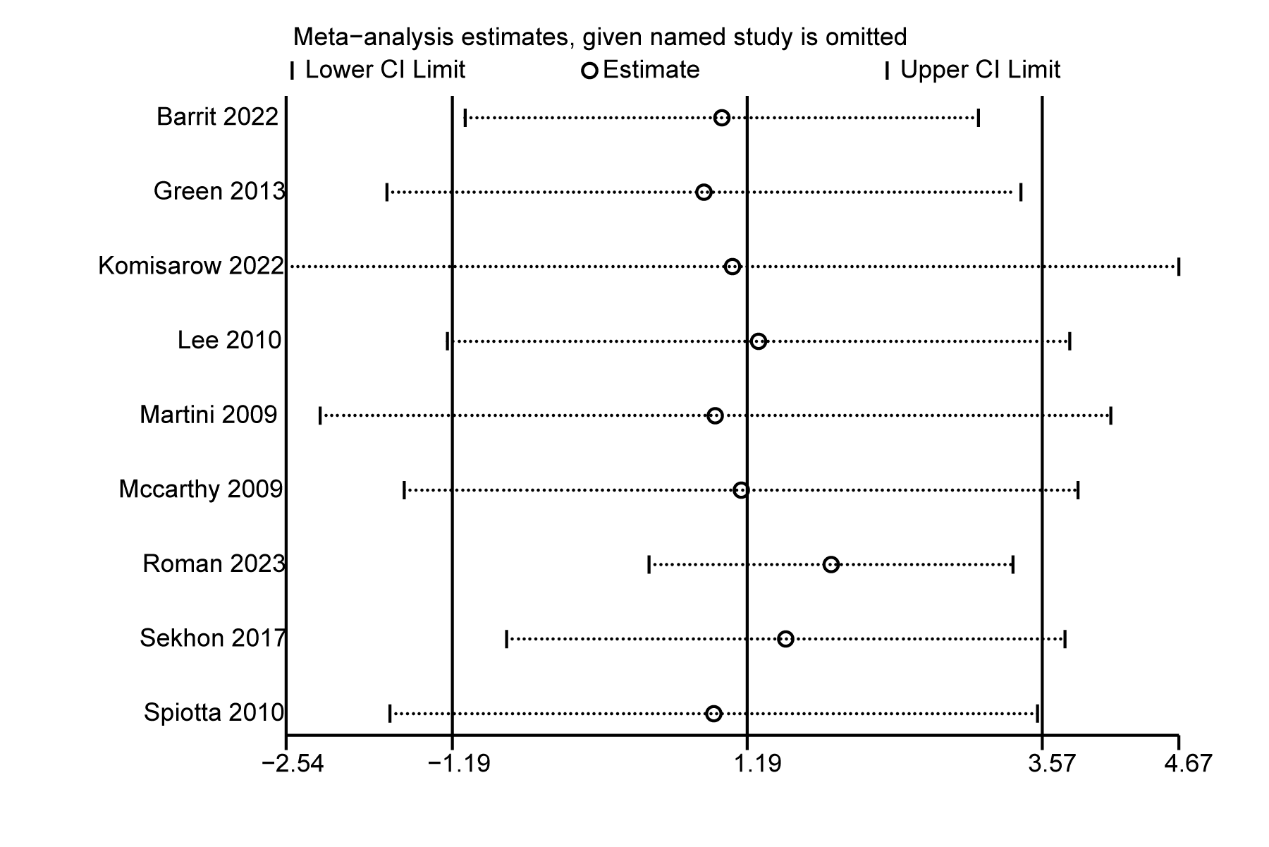


Figure 7: Sensitivity analysis for length of hospital stay


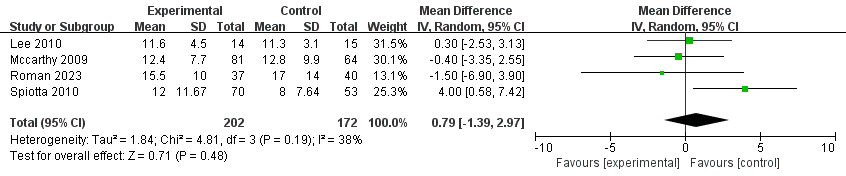


Figure 8: Sensitivity analysis for length of ICU stay by excluding studies reported length of stay data in the form of median and IQR


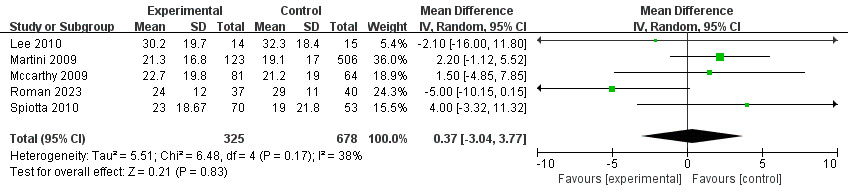
 Figure 9: Sensitivity analysis for length of hospital stay by excluding studies reported length of stay data in the form of median and IQR
