# Supplementary material for: Brain tissue oxygen pressure combined with intracranial pressure monitoring may improve clinical outcomes for patients with severe traumatic brain injury: a systemic review and meta-analysis
Source: PeerJ. 2024 Oct 8;12:e18086. doi: 10.7717/peerj.18086 (PMC11468803; doi:10.7717/peerj.18086)
Supplement: Supplemental Information 5 [file peerj-12-18086-s005.docx]

**Supplementary Material 4:** Trial sequential analysis


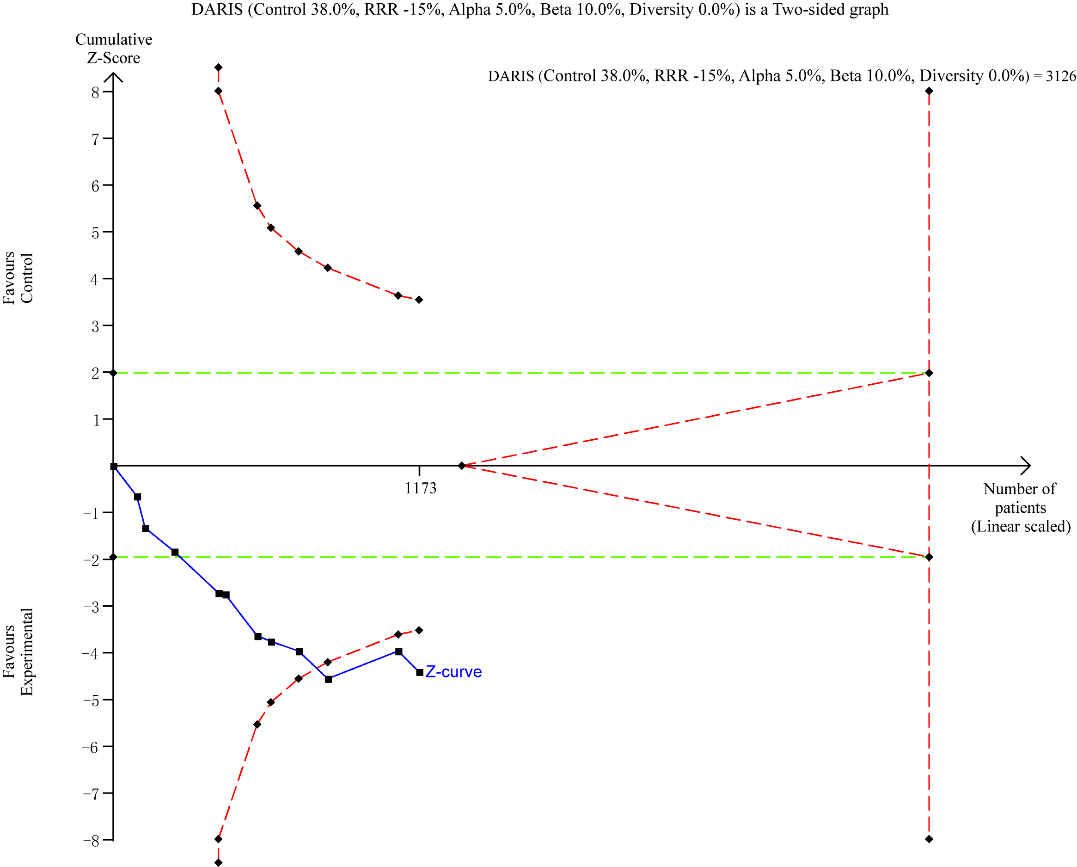


Figure 1: Trial sequential analysis for favourable neurological outcome at 6 months


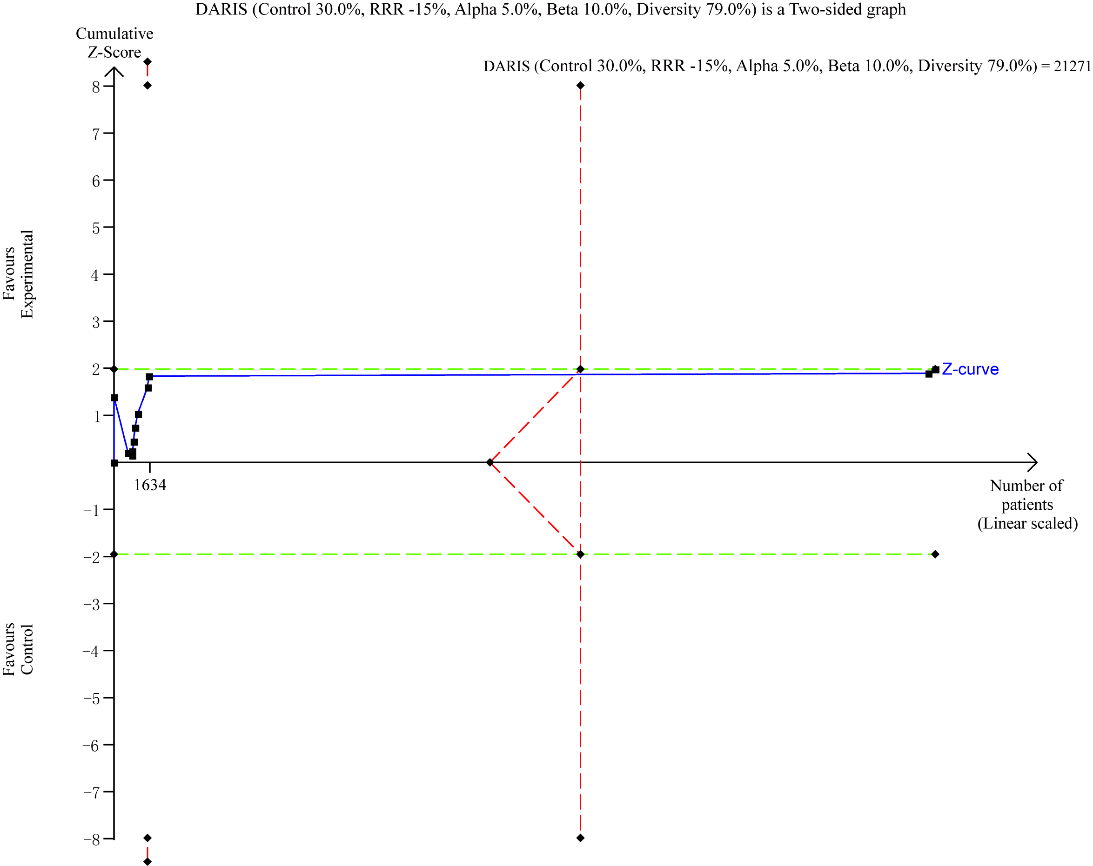


Figure 2: Trial sequential analysis for in-hospital mortality


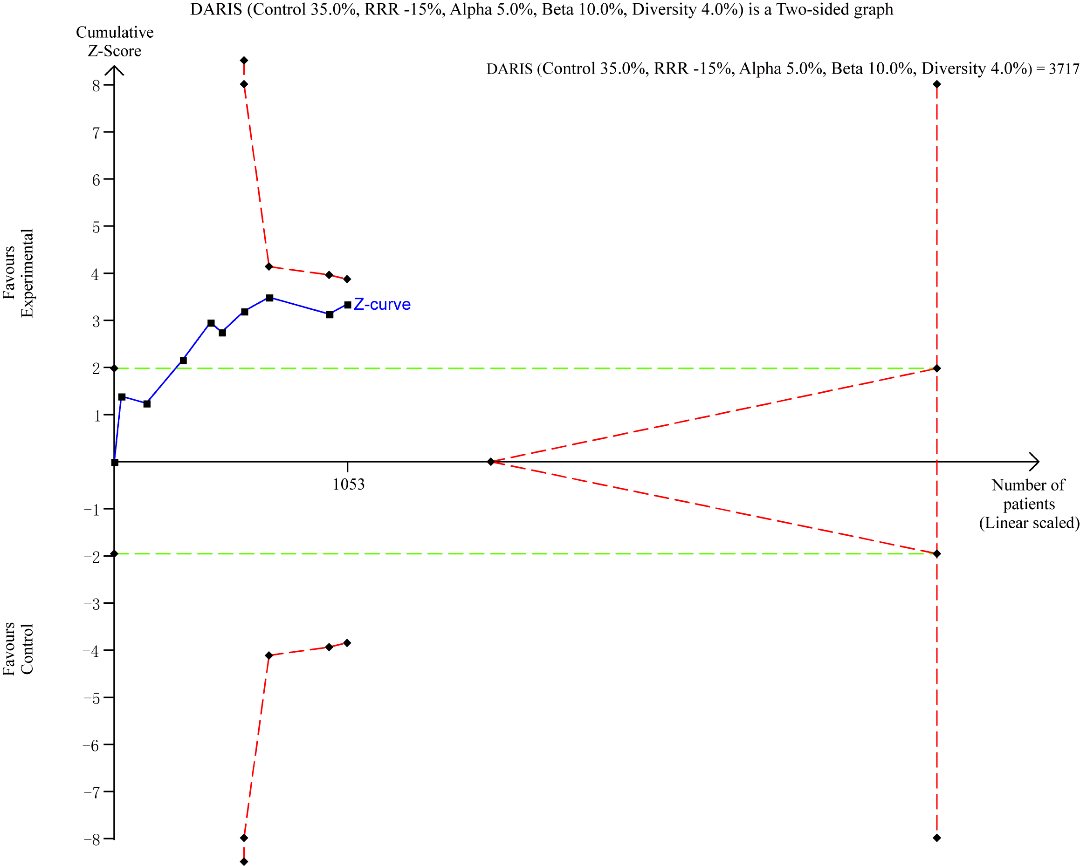


Figure 3: Trial sequential analysis for long-term mortality


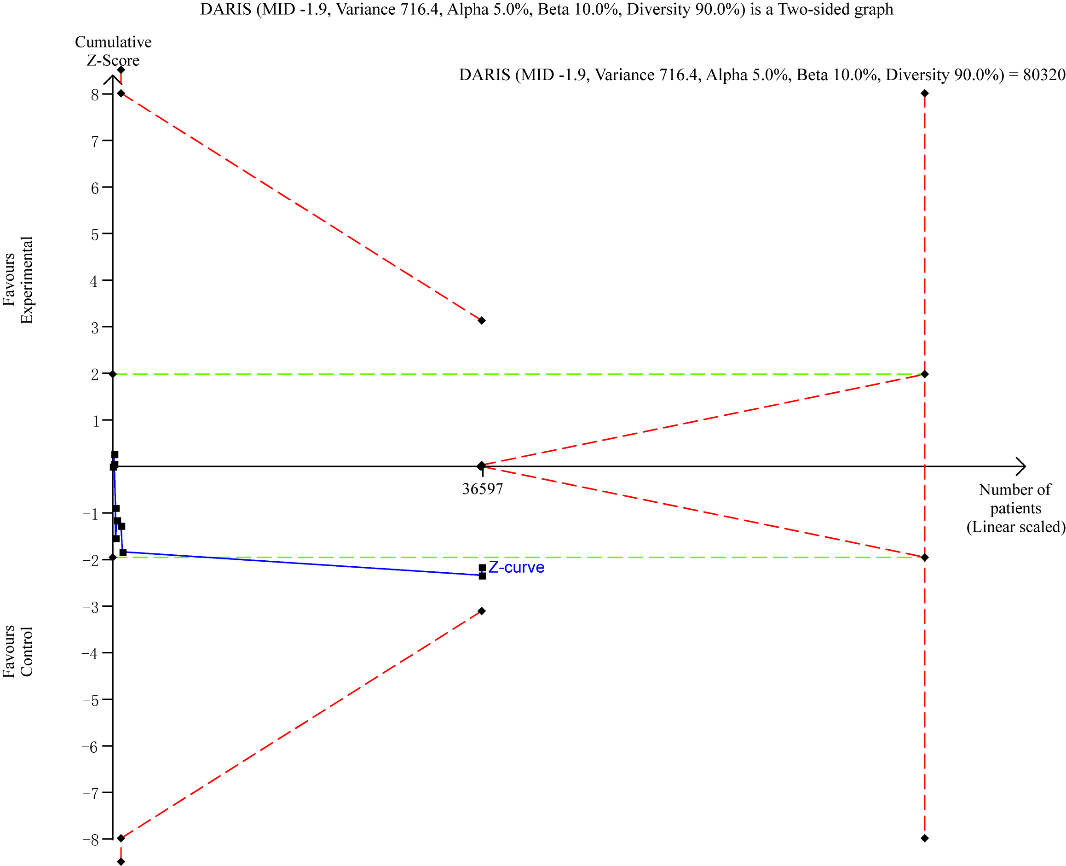


Figure 4: Trial sequential analysis for length of stay in intensive care unit


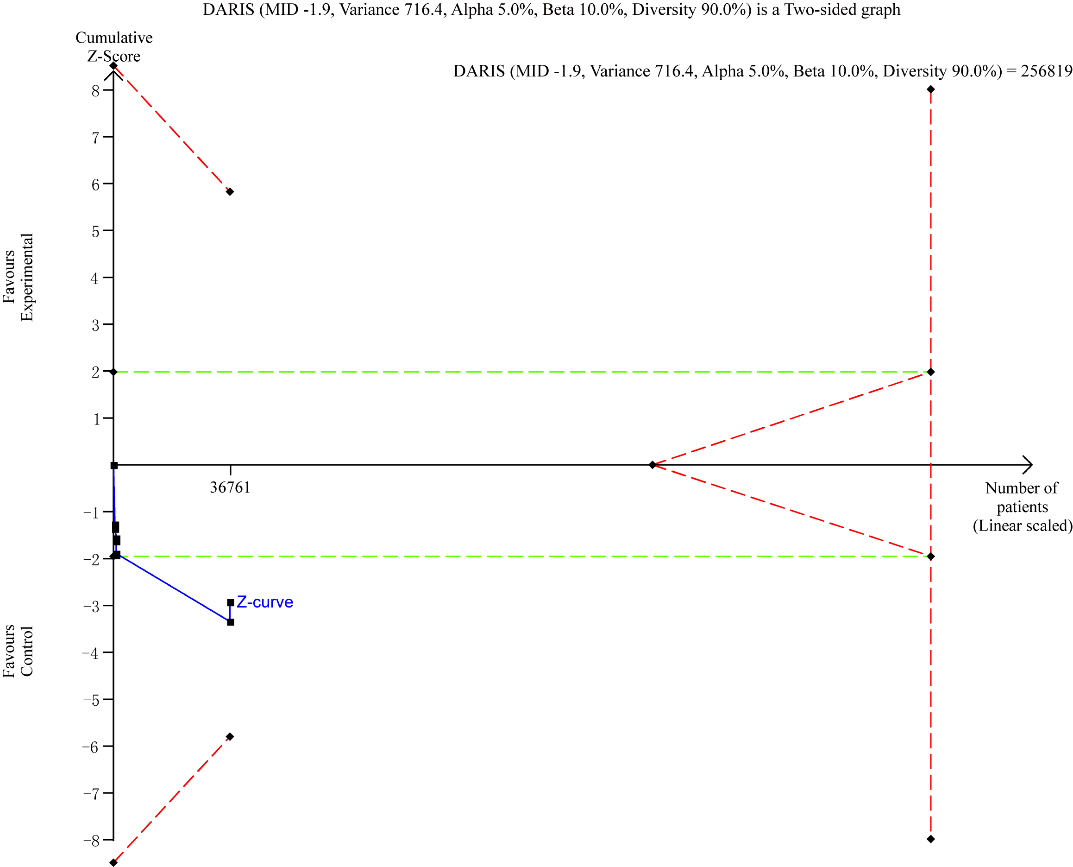


Figure 5: Trial sequential analysis for length of stay in hospital
